# An Interaction Path of Mothers’ and Preschoolers’ Food- and Physical Activity-Related Aspects in Disadvantaged Sicilian Urban Areas †

**DOI:** 10.3390/ijerph18062875

**Published:** 2021-03-11

**Authors:** Garden Tabacchi, Luca Petrigna, Giuseppe Battaglia, Giovanni Navarra, Antonio Palma, Marianna Bellafiore

**Affiliations:** Sport and Exercise Sciences Research Unit, Department of Psychology, Educational Science and Human Movement, University of Palermo, Via Pascoli 6, 90144 Palermo, Italy; tabacchi.garden@libero.it (G.T.); giuseppe.battaglia@unipa.it (G.B.); giovanniangelo.navarra@gmail.com (G.N.); antonio.palma@unipa.it (A.P.); marianna.bellafiore@unipa.it (M.B.)

**Keywords:** mothers, preschoolers, food habits, physical activity, association, disadvantage

## Abstract

*Background*: The relationship between mothers and their children’s lifestyle is still unclear, especially in disadvantaged areas. Consequently, the study aims to identify a path explaining the extent to which maternal eating habits and physical activity (PA) level predict food-related aspects, PA practice and Quotient of Gross Motor Development (QGMD) in preschoolers from disadvantaged urban areas. *Methods*: In this cross-sectional study, a total of 79 dyads of mothers and children were recruited from kindergartens. Information related to family socio-demographic aspects, mothers’ and children’s dietary intake frequencies and PA/sedentariness, mothers’ weight and height, mothers’ perception on children’s food intake, and children’s food literacy (FL) was collected with a questionnaire and the Food Literacy Assessment Tool (preschool-FLAT), while gross-motor skills were measured with the Test of Gross Motor Development (TGMD); weight and height of children were directly collected. *Results*: Associations were found between mothers’ and children’s food habits; mothers’ and children’s fruit/vegetables consumption, and intake of the other items; mothers’ education or PA level and children’s FL; mothers’ PA or sedentariness and children’s QGMD; mothers’ BMI and food habits and children’s BMI; education and food habits. *Conclusions*: These findings can be useful to plan effective interventions targeted both to preschoolers and their mothers of disadvantaged urban areas for promoting healthy lifestyles, which have become increasingly difficult to achieve during COVID-19 pandemic.

## 1. Introduction

Several studies have reported that pre-school children present a high and increasing prevalence of overweight and obesity [1]. The influence of parents on their children’s lifestyle since the pre-school age has a fundamental role in the prevention and management of obesity and other chronic conditions later in life through the monitoring of food choices [2], sedentary behavior and physical activity (PA) practiced [3].

The quality of children’s diet, which is related to the choice of healthy food, is usually influenced by parents’ food choices [4]. Remarkably, the eating behaviors and habits of mothers are positively correlated to their sons’ nutritional knowledge and food habits [5], and this could be due to the amount of time spent with them. Children’s diet is also affected by the parents’ perception of their children’s body weight status [6]; this association is also mediated by the parents’ body mass index (BMI), and remains throughout childhood [7].

Regarding the sedentary behaviors, an association between parental and child (aged one through four years) television viewing was noted [8] and the amount of time spent viewing TV/video significantly increased the risk of overweight [9]. With respect to PA, there is a correlation between parents’ and children’s PA with more active parents tending to have active children [10]. In particular, mothers importantly enhance PA practice and reduce sedentary time of their children in preschool age [11]. An increase in PA has been reported to be significantly associated with an improved level of gross-motor skills regardless of the state of body weight in preschool children [12].

Lifestyles and behaviors are often determined by the family socio-economic (SE) status and cultural aspects. In developed countries, different studies demonstrated the effect of SE inequalities in dietary patterns across youth [13]. E.g., higher parental SE position is associated with more healthy dietary habits among adolescents [14]; higher mother’s education is an important predictor of better child and adolescent food choices and habits [15]. It often happens that the culture of people in such low SE urban areas is that the more you eat the more you are healthy, and they don’t understand what “healthy” means and what are the healthy foods and behavior choices; thus, they are not able to convey to their children correct information on correct food habits; moreover, parents belonging to low SE status don’t have enough money to buy the food of higher quality. Inconsistent results have been shown for children and adolescents in a recent review analyzing the associations between SE determinants and PA behavior across the life course; no associations were found out in the 3–6 age range, while opposite results were shown for schoolchildren and adolescents [16]. This could be likely due to the multi-dimensional nature of PA and SE status that encompasses considerable methodological measurement concerns [17]. The influence of low SE urban areas in our region could be seen for example in the lacking of places dedicated to free sport sessions, often because of lacking of public facilities and services, so that children can practice low PA; some areas have also hygienically deprived open spaces (amounts of garbage along the roads and dirty streets) where children cannot play; it also happens that parents have not often the economic opportunities of taking their children to practice some activities in gyms or similar closed places.

Moreover, healthy choices and correct lifestyles are related to “health literacy”, a concept that promotes individual empowerment going beyond the idea of health education and behavior-oriented communication, and is referred to environmental, political and social aspects determining health [18]. Health literacy develops in early stages of life [19] and seems associated with important health outcomes [20]. Higher parents’ health literacy seems to be negatively correlated with BMI of parents themselves [21] and affects the BMI of their children [22]. Low health literacy may be a risk factor for using less reliable sources of health information and for selecting more unsafe weight control strategies for children [23]. The recent concept of “food literacy” derives from health literacy, and is referred to food production, procurement, preparation, processing, packaging, and labeling to food choice and consumption [24], and similarly starts developing since preschool age.

As so far exposed, some data exist in the literature analyzing how single parental lifestyle factors can be determinants of preschoolers’ food habits/behaviors and PA, little information is present about comprehensive relationship models. Some aspects related to the relationships between mothers and their children were previously presented during “The 3rd International Electronic Conference on Environmental Research and Public Health —Public Health Issues in the Context of the COVID-19 Pandemic” [25] and these will be further investigated in the present study. Thus, the present study aims to investigate the extent to which maternal food habits and PA level predict food-related aspects, PA practice and gross-motor development in pre-school children attending kindergartens from low SE level urban areas and provide a comprehensive interaction path.

## 2. Material and Methods

### 2.1. Study Design and Participants

This is a cross-sectional study conducted between February and June 2016 within the *Training-to-Health* Project [26]. The study participants were recruited within kindergartens placed in disadvantaged boundaries of the Palermo City Council. The SE level of the school areas was evaluated through the “index of socio-economic disadvantage”, measured based on of four indicators of deprivation in the 55 different city districts [27].

Out of the 100 parents that were invited to join the study and provided with detailed information sheets on the project, 87 accepted by signing informed consents (response rate 87.0%) and they were all mothers. Those who refused reported they did not wish to provide private information about themselves and their children. They were asked to self-compile a properly developed questionnaire; six responses were excluded as they were not completed, and for two children data were not collected due to school absence and incomplete assessment, respectively. Finally, data were obtained for a total of 79 dyads of mothers and children aged 3–6 years.

### 2.2. Data Collection and Outcomes

The questionnaire administered to mothers was properly developed by the project team and included different items to assess lifestyle and socio-demographic aspects both on mothers and their children.

Mothers’ and children’s frequencies of dietary intakes were assessed, i.e., fruit and vegetable intake, carbonated/sugared drinks consumption, sweet and savoury snacks intake, ready meals intake, breakfast frequency. The frequency options differed depending on the item: for fruit and vegetables intake six options were proposed (never, less than once/week, some time/week, once/day, 2–4 times/day, 5 or more times/day); for carbonated/sugared drinks five options (never, less than once/week, some time/week, once/day, more than once/day); for sweet and savoury snacks and breakfast frequency five options were proposed (never, less than 3 times/week, 3–4 times/week, 5–6 times/week, every day); for ready meals, three options were envisaged (never, sometime, every day). From these five items, an overall score of healthy diet (referred to as “food habits score” in this paper) was obtained by summing up the scores of the single food item. The final score ranged from 0 to 14, with higher scores being attributed to better food habits. For the study, the different categories of intake were grouped in binary variables, that encompassed low and medium/high frequencies.

Mothers were also asked about the duration of breastfeeding of their children, with four frequency options (six or more months, 3–5 months, less than 3 months, never).

Physical activity and sedentariness were assessed for mothers and children by asking the number of days in a week when they performed at least one hour of PA (they could choose between 7, 5–6, 3–4, 1–2 and 0 days), and the daily hours of standing in front of video/PC (this was an open question).

Mothers were also asked about their perceived amount of children’s food intake (little, fair, too much) and perceived children’s weight status (under, normal, a little over, or too much overweight).

Mothers self-reported their weight and height, and, after BMI was calculated, classes of weight status were obtained according to the BMI cut-offs from the CDC [28]. Weight and height of children were measured within the schools by operators of the project team that were previously trained and standardized, and categories of weight status were assessed through the BMI cut-off values according to gender and age from Cole et al. 2000 [29].

Information on mothers’ education level (none, primary, secondary low, secondary high, university) and occupation (none, part-time or full-time) were collected.

The preschool-Food Literacy Assessment Tool (FLAT), a tool developed and validated by the project team [30], was used to assess the food literacy (FL) score of children. It was administered by the project team for a total of ten hours in five sessions, which consisted of a brief oral session providing information on the food topics, and a practical session ending with an evaluation sheet compiled by the children. The results of the FL assessment were FL scores ranging from 0 to 20 (where 0 indicates no FL and 20 indicates high FL) that were obtained by the sum of the scores of the single items of each of the four food domains; in fact, a 5-point scale (from 0 to 4) according to Likert was possible for each domain, and the sum of the single domain score provided a total maximum value of 20.

The Quotient of Gross Motor Development (QGMD), encompassing locomotor abilities and object control skills, was also assessed, by using the Italian version of gross motor development test [31]. This test envisages that subjects run 15 m as fast as possible, gallop for 10 m, hop on one leg for 5 m, jump forward, do a long jump, and take little jumps forward and laterally, catch a ball with a tennis racket, bounce off the ball, catch a ball, kick the ball running, and throw a ball with the hand [32].

### 2.3. Statistical Analysis

Sample characteristics were calculated in number and percentages, and means and standard deviations (SD) were used for normally distributed continuous data. Differences between mothers’ and children’s intake frequencies were estimated through paired T-test for the food habit score, and through chi-squared test for the single food items. Chi-squared test was used also to estimate the difference between perceived and measured variables. To assess the associations between food-related aspects a logistic regression was performed.

To evaluate a path of the relationships between mothers’ food habits or PA practice and food habits, food literacy and PA in their children, a Structural Equation Modeling (SEM) analysis was performed. This technique combines factor analysis and multiple regression analysis to modeling the interactions between variables [33]. A model with only observed variables is proposed, with each item used as a separate construct indicator; this analysis represents a detailed analysis for SEM [34], useful for assessing psychometric properties of every single item.

Both non-standardized and standardized beta coefficients were estimated that reflect the degree of change in the outcome variable associated with a standard deviation change in the predictor. These standardized regression coefficients allow to evaluate relationships in those studies where different units of measure have been used [35].

To determine how adequately the model fit to the data, multiple fit indices were used. The comparative fit index (CFI), the Tucker-Lewis Index (TLI), the root-mean square error of approximation (RMSEA), the standardized root mean squared residual (SRMR), the relative χ^2^. A value ≥ 0.9 is considered a good fit for the CFI and TLI. The RMSEA tests the fit of the model to the covariance matrix; a value of 0.05–0.08 is considered as an acceptable fit and < 0.05 as a good fit. The SRMR is the square root of the discrepancy between the sample covariance matrix and the model covariance matrix and has an acceptable fit value of 0.08 or less. The relative χ^2^ is the χ^2^ divided by the degrees of freedom and it was used because is thought to be less dependent on sample size; smaller values indicate a better fit with a value of 3 or less considered as acceptable [36,37,38,39,40].

Statistical significance was accepted at *p* < 0.05. The STATA/MP 12.1 software (StataCorp LP, College Station, TX, USA) was used for the statistical analysis, with the “sem” command to perform the SEM analysis.

## 3. Results

### 3.1. Sample Characteristics

Table 1 shows the characteristics of the 79 dyads of mothers and children. Three-quarters of the mothers had low education, and around half of them were not occupied (46.8%).

### 3.2. Interactions Path

The overall model fitted the observed data well, as shown by the fit indices of the SEM analysis in Table 2: CFI 0.987, TLI 0.980, RMSEA 0.026, SRMR 0.035, χ^2^/df 1.37 *p* < 0.001.

**Figure 1:**
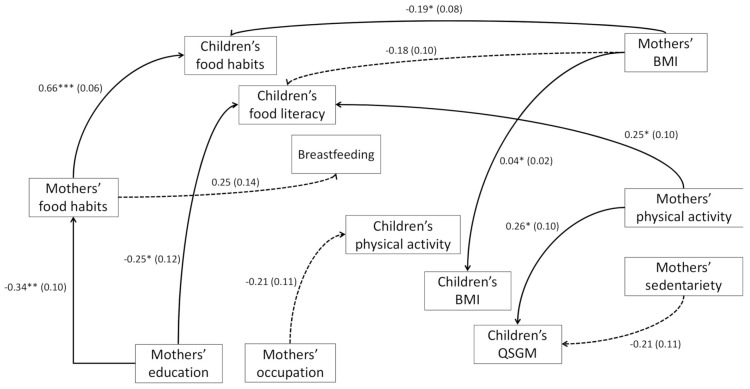
Interactions between maternal and their children food and PA habits. Note: Path coefficients are presented as standardised regression weights and standard errors in parenthesis. Only significant relationships are showed; asterisks indicate the significance level (* *p*-value < 0.05; ** *p*-value < 0.01; *** *p*-value < 0.001). The dashed lines represent relationships with a significance level slightly above 0.05 (mothers’ BMI/children’s FL score *p* = 0.080; mothers’ occupation/children’s PA *p* = 0.061; mothers’ sedentariness/QSGM *p* = 0.053; mothers’ food habits/breastfeeding *p* = 0.073). BMI: body mass index; QGMD: quotient of gross motor development.

The possible path of the relationship between mothers’ food habits/PA and children’s food and PA aspects is shown in Figure 1.

The path shows that mothers’ food habits are highly associated with children’s food habits (beta = 0.66, *p* < 0.001). A non-significant relationship is shown between mothers’ food habits and breastfeeding, even though significance level is very close to *p* = 0.05.

Children’s FL seems to be influenced by both mothers’ education (beta −0.25, *p* < 0.05) and PA level (beta 0.25, *p* < 0.05), with higher education being correlated to a higher FL level, and a higher PA level being associated to an increased FL level.

Children’s PA is not influenced by their mothers’ PA practice or sedentariness, but it seems that a trend could be present (even though no statistical significance is shown) with mothers’ occupation; e.g., mothers’ having no job contributes to a low children’s PA level. Mothers’ PA and sedentariness result to be possible predictors of children’s QGMD (beta 0.26, *p* < 0.05; and −0.21, *p* < 0.1, respectively), with mothers’ practicing higher levels of PA or being less sedentary influencing higher children’s QGMD.

A higher mothers’ BMI is significantly correlated to incorrect food habits (beta −0.19, *p* < 0.05) and to higher BMI of their children (beta 0.04, *p* < 0.05). In the model, correlations between mothers’ lifestyles have also been included, but only a higher education was shown to influence better food habits (beta −0.34, *p* < 0.05).

### 3.3. Associations between Mothers’ and Children’s Food Intake Frequencies

With regard to the food-related aspects, mothers’ food habits mean score was 10.2 (SD 2.42), while the children’s one was 9.4 (SD 2.43) (Table 3). A quite high proportion of both mothers and children did not reach the five portions of fruit and vegetables per day (41.8% and 46.8%, respectively); sugar and carbonated drinks were consumed one or more times/day in a quarter of mothers (24.0%) and in more than one-third of children (36.7%); more than a half of children, moreover, consume confectioned sweet and savoury snacks every day or many times during a week (54.4%); breakfast is consumed frequently by both mothers and children (Table 3). Children of mothers consuming high amounts of fruit and vegetables are significantly more prone to having high intakes (OR 16.2, 95% CI 4.68–59.77); this occurs also for the other items (Table 3).

### 3.4. Mothers’ Perceptions

No mother perceived her own child as obese, while 11.4% were measured and classified as obese; only 2.5% perceived their children as overweight, while 19.0% were measured and classified as overweight; those mothers perceiving their children as underweight were the 10.3%, a percentage that corresponded to that of underweight measured children; normal weight perceived children were the major part 87.4%, while only around 60% were measures as normal weight (*p* < 0.01) (Table 4).

Mothers did not correctly perceive the PA practicing (*p* < 0.05), with around 50% declaring that their children practiced little PA (vs. 72%), 47% perceiving sufficient PA *vs* 13% of children practicing fair amount of PA, and 4% *vs* 15% regarding the perception and measured higher levels of PA, respectively (Table 4).

A total of 19.2% of mothers perceived a low intake of their children, while only 5.1% think that their children have a high amount of food (Table 4). Since data on measured food intake of children is not available in this study, a comparison is not possible.

## 4. Discussion

This study provides a path of the interactions between mothers’ and their children’s food and PA habits. As from the study design, the selected schools were recruited mainly in low SE areas of the town, where mothers were mainly housewives and low-educated.

From the path, it appears that mothers’ food habits are clearly good predictors of children’s food habits, as confirmed in other studies [41,42], and similarly mothers’ BMI influences food choices of children, with higher BMI being associated to worse food habits. As expected, mothers’ BMI is correlated to children’s BMI [43]. This condition is evident in Southern Italy throughout growing age until adolescence, with overweight/obese parents predicting significantly their children’s overweight/obesity [44].

With regard to the specific food consumption frequencies, it occurs that still high percentages of children take incorrect food choices, as they do not eat enough fruit and vegetables, drink too frequently sugared carbonated drinks, and eat daily sweet and savory snacks and ready meals, in a such early phase of age [45]. Around 90% of children have regular breakfast from 5 to 7 days a week, and this is an encouraging fact, especially compared to some children showing lower frequencies of breakfast consumptions [46]; the percentage found in the present work is quite in line with the Dortmund Nutritional and Anthropometric Longitudinally Designed (DONALD) study, stating that around 86% of children aged 2–5 regularly have breakfast [47].

The logistic regression analysis of the present study showed further details on the relationships between mothers’ and their children’s food habits. In general, significant associations with high ORs were found out between mothers and their children’s food habits, with virtuous mothers having higher probability of educating virtuous children. This is confirmed in previous findings stating that mothers influence the fruits and vegetables intake of their children, as they are the direct responsible for purchase decisions, for monitoring and reminding their children’s food intake, for recommending healthy food intake before allowing snack or junk food consumption [41,48].

With regard to the children’s FL, which is an important component of health literacy, the study shows that it depends on mothers’ education and PA practice. It has to be evidenced that FL involves both motor ability components and basic literacy skills, which on their turn include key components such as gross and fine motor abilities, and oral language, alphabetic code and print understanding and use [49]. Thus, it is suggested that children from high-educated mothers have more incentives and motivations in developing better motor and emergent literacy skills [50]. Mother’s occupation does not seem to influence children food-related aspects, and this is apparently in contrast with one study where the working mother or father was significantly associated with higher school canteen attendance compared to children from non-working parents; in the same study no association was present between occupational status (high, medium or low) or household wealth index of the mother/father and canteen attendance of their pre-school children [51]. The relationship is anyway confused, as in that study mother and father were not distinguished, then it can be hypothesized also that the food-related aspect could generally more strictly related to father’s occupation.

Mother’s PA practice is a predictor of children’s QGMD in this study, and similarly a possible trend has been observed for mother’s sedentariness influencing the QGMD; unfortunately, no studies in the literature exist that can confirm this finding. Anyway, the literature suggests that parent’s role is important to promote their children’s motor competences and skills [52] and probably, a mother that practices more PA, influences her children’s habits to a more active childhood.

Even though the authors were expecting a positive association between mother’s and children’s PA, the practice of PA in mothers was not an independent predictor of their children’s PA, as stated in some literature studies [53,54,55], but in contrast with another one [56].

The mothers’ perceptions of their children’s weight were not correct (*p* < 0.01). The authors initially believed that this condition could have been typical of the tradition of Southern Italy, where mothers consider a child “healthy” if he is chubby and well-fed. However, the international literature confirms that a significant percentage of children’s excess weight is not perceived by the parents, and this perception is associated with differences in dietary habits, PA and sedentariness [57]; worldwide adjusted effect sizes reveal half of parents underestimating their overweight/obese children’s weight and a minority underestimating children’s normal weight, independently from geographical area and socioeconomic level, as reported in a recent meta-analysis [58]. Furthermore, this is confirmed in a review targeted at preschoolers, where authors report that parents underestimate their child’s overweight status or are not aware of the risks associated with overweight [59].

Due to the lack of information in the literature regarding the perceptions of parents on the PA practice and on the intake, it results difficult to comment the results found in this work.

One strength of the present work is that most data were accurately collected, indeed, information on food literacy is considered highly precise as the validated preschool-FLAT was used [30]. Even though the questionnaire administered to mothers was not validated, since a gold standard for comparison does not exist in the literature, the authors tried to include questions (with proper frequency options) useful to collect the information on food intake and PA both of mothers and children needed to evaluate the correlations that explain the influence of mothers on their children’s lifestyles.

A limitation of the present work is that the data were collected in 2016. However, we believe that few years could not significantly change this inter-relationship pattern, since modifications of behaviors take long time to occur, especially in disadvantaged contexts where facilities take time to be provided and certain culture is difficult to eradicate. Life style behaviors need time and effort to be changed, and this should be indeed one of the objectives of the local actions targeted to adult and young population groups. Another limit of this study is the lack of information about the presence of chronic metabolic and cardiovascular diseases in the study group. Future studies could be addressed to deepen the relationship between chronic parents’ disease and food behaviors of their children.

Data collected for PA of mothers and children were self-reported in the used questionnaires, and then less accurate than collected by accelerometers or other tools for accurate measures [60]. Moreover, for mothers’ anthropometric data the information was self-reported. These aspects could limit the accuracy of results.

Another limitation of the present work is that data were collected only for mothers, since only mothers signed the informed consent and were present the day of recruitment and data collection. On one hand it is known that mothers in the family are the main responsible for calibrating the food provision to their children, struggling to eat healthy, consume ethically, and feed them properly [61]. On the other hand, a new role of fathers in the last decade has been delineated as direct participants in food provision for their children [62,63]. Despite the importance of these new findings, the authors believe that the SE context considered for the present study is still characterized by a traditional family management, with mothers assuming prevalently domestic food responsibilities and being more involved in shaping their children’s lifestyles. Furthermore, in this period, to know the role of the mothers on their children health is even more important considering that the Sars-CoV-2, the so-called COVID-19 is the cause of a decrease in quality of PA and an increase in sitting time, of an unhealthy diet (in terms of type of food, eating out of control, snacks between meals and number of main meals) [64], of the mental well-being and psychological health [65] and that the anxious mood due to the virus impact also the worsen the eating habits [66]. Thus, results from this study could be considered reliable and suggest that interventions to prevent incorrect food habits and sedentariness in similar SE contexts should be targeted to preschoolers and their mothers.

## 5. Conclusions

This study identified a comprehensive path of the relationships between mothers’ and children’s lifestyles in low SE urban areas. According to this pattern, children’s food habits and food literacy are significantly influenced by mothers’ food habits, BMI and education, while mothers’ occupation seems not to be an important predictor. The practice of PA in mothers is not an independent predictor of their children’s PA, but it influences their gross motor quotient. Results of the present study can be currently useful to plan effective interventions targeted to mothers from disadvantaged urban areas for promoting healthy lifestyles, which have become increasingly difficult to achieve during COVID-19 pandemic.

## Figures and Tables

**Table 1:** Characteristics of the 79 dyads of mothers and children participating in the Training-to-Health Project.

Parent	N.	%	Child	N.	%
Gender			Gender		
Male	0	0	Male	42	53.2
Female	79	100.0	Female	37	46.8
Education			Age (months)		
Primary	27	34.2	≤47	16	20.2
Secondary	33	41.8	48–59	41	51.9
Degree	19	24.0	≥60	22	27.9
Occupation	79		Schools SEE		
Full-time	19	24.1	Low	71	89.9
Part-time	23	29.1	High	8	10.1
Non occupied	37	46.8			
Weight status			Weight status		
Under	3	3.8	Under	8	10.1
Normal	47	59.5	Normal	47	59.5
Over	24	30.4	Over	15	19.0
Obese	5	6.3	Obese	9	11.4
Video/PC sitting time (h/day)			Video/PC sitting time (h/day)		
≤2	47	63.5	≤2	46	59.7
>2	27	36.5	>2	31	40.3
	**mean**	**SD**		**mean**	**SD**
Height (m)	1.6	0.06	Age (mo)	56.0	8.90
Weight (kg)	65.3	10.86	Height (m)	1.07	0.06
BMI (kg/m^2^)	24.2	3.71	Weight (kg)	19.2	2.49
PA (h/week)	2.2	1.91	BMI (kg/m^2^)	16.8	1.95
			PA (h/week)	2.4	1.79
			QGMD	123.0	15.39
			FL score	12.5	4.56
			Breastfeeding (mo)	2.7	1.96

BMI: Body Mass Index; PA: Physical Activity; QGMD: Quotient of Gross Motor Development; FL: Food Literacy.

**Table 2:** Fit indices of the Structural Equation Modeling analysis.

Confirmatory Factor Index (CFI)	0.987
Tucker-Lewis Index (TLI)	0.980
Root Mean Square Error of Approximation (RMSEA)	0.026
Standardised root mean squared residual (SRMR)	0.035
Chi-square/df, *p*-value	1.37, *p* < 0.001

**Table 3:** Food-related aspects of the 79 dyads of parents and children participating in the Training-to-Health Project.

	Parent		Child		Odds Ratio (95% CI) ^§§^
	Mean	SD	Mean	SD	
Food habits score ^§^***	10.2	2.42	9.4	2.43	
	**N.**	%	**N.**	%	
Fruit/vegetables ***					
2–5 times/day	46	58.2	42	53.2	16.2 (4.68–59.77)
<2 times/day	33	41.8	37	46.8	
Sugared/carbonated drinks ***					
never or some time/week	60	75.9	50	63.3	34.0 (6.34–322.87)
1 or more times/day	19	24.1	29	36.7	
Snacks ***					
never or <3times/week	60	75.9	52	65.8	11.1 (2.25–104.78)
3–7 times/week	19	24.1	27	34.2	
Ready meals ***					
never	46	58.2	49	62.0	25.6 (6.65–107.04)
sometime/everyday	33	41.8	30	38.0	
Breakfast frequency ***					
5–7 times/week	72	91.1	79	88.6	17.9 (2.17–147.90)
<5 times/week	14	17.8	14	17.7	

^§^ Difference estimated through paired T-test. ^§§^ Associations estimated through logistic regression analysis. *** *p* < 0.001.

**Table 4:** Parent’s perceptions frequencies of children’s weight status, intake and PA.

Perceived Items	n	%	Measured Items	n	%
Perceived weight ^§^**			Measured weight		
Under	8	10.1	Under	8	10.1
Normal	69	87.4	Normal	47	59.5
Over	2	2.5	Over	15	19.0
Obese	0	0	Obese	9	11.4
Perceived PA ^§^*			Measured PA		
Little	39	49.4	0–1.5 h/week	57	72.1
Fair	37	46.8	2–4 h/week	10	12.7
Too much	3	3.8	>4 h/week	12	15.2
Perceived intake					
Little	15	19.2			
Fair	59	75.6			
Too much	4	5.1			

^§^ The difference between perceived and measured variables was estimated through chi-square test. * *p* < 0.05; ** *p* < 0.01.

## Data Availability

Data used during the current study are available from the corresponding author on reasonable request.
